# Anisotropic conductivity tensor imaging for transcranial direct current stimulation (tDCS) using magnetic resonance diffusion tensor imaging (MR-DTI)

**DOI:** 10.1371/journal.pone.0197063

**Published:** 2018-05-15

**Authors:** Mun Bae Lee, Hyung Joong Kim, Eung Je Woo, Oh In Kwon

**Affiliations:** 1 Department of Mathematics, Konkuk University, Seoul, Korea; 2 Department of Biomedical Engineering, Kyung Hee University, Seoul, Korea; Shanghai Mental Health Center, CHINA

## Abstract

Transcranial direct current stimulation (tDCS) is a widely used non-invasive brain stimulation technique by applying low-frequency weak direct current *via* electrodes attached on the head. The tDCS using a fixed current between 1 and 2 mA has relied on computational modelings to achieve optimal stimulation effects. Recently, by measuring the tDCS current induced magnetic field using an MRI scanner, the internal current pathway has been successfully recovered. However, up to now, there is no technique to visualize electrical properties including the electrical anisotropic conductivity, effective extracellular ion-concentration, and electric field using only the tDCS current *in-vivo*. By measuring the apparent diffusion coefficient (ADC) and the magnetic flux density induced by the tDCS, we propose a method to visualize the electrical properties. We reconstruct the scale parameter, which connects the anisotropic conductivity tensor to the diffusion tensor of water molecules, by introducing a repetitive scheme called the diffusion tensor **J**-substitution algorithm using the recovered current density and the measured ADCs. We investigate the proposed method to explain why the iterative scheme converges to the internal conductivity. We verified the proposed method with an anesthetized canine brain to visualize electrical properties including the electrical properties by tDCS current.

## Introduction

Transcranial direct current stimulation (tDCS) was originally developed for brain injuries such as strokes or major depressive disorder. tDCS has reported to be effective in treating a number of neurological and psychiatric disorders. Recent findings show novel possibilities by confirming significant changes in the pain-related neural networks among patients with chronic pain [1, 2]. tDCS can improve performance on a variety of cognitive tasks in humans, in both healthy and in patients, despite its exact mode of action remains unclear [3–5]. Further researches need to develop translational studies to better understand how tDCS improves memory, a necessary condition before it can be used as a therapeutic tool. For the long-term heroin-addicted subjects, the tDCS over bilateral frontal-parietal-temporal (FPT) region has been shown to significantly reduce subjective craving score induced by heroin queues of heroin addicts [6]. tDCS is a therapeutic modality as an add-on treatment that showed promising results for major depressive disorder [7–9].

tDCS typically uses the intensity of injection current between 1-2 mA for a duration of up to 20 minutes. However, it is insufficient to directly estimate the internal electrical properties (current density and/or conductivity) when tDCS operates by sending low direct current through the attached electrodes on the head. Up to now, computational modeling and simulation techniques have been used to characterize and understand the effects of tDCS, including electrode size, the number of anode and cathode locations, current density, and electrical field.

Using an MRI scanner, combining with the external current injections, electrical properties (current density and/or conductivity distribution) have been widely studied [10, 11]. Recently, direct visualization of magnetic field changes by tDCS current was studied [12]. Magnetic resonance electrical impedance tomography (MREIT) technique aims to visualize the internal current density and the isotropic conductivity by measuring only one component of magnetic flux density using an MRI scanner [11, 13]. Using MREIT modality, for the human brain cases, the internal current density distribution in the human brain from the measured magnetic flux density by tDCS current was studied [14]. However, no direct techniques exist to visualize the electrical properties including anisotropic conductivity tensor, electric field, and apparent concentration of ions by tDCS current. To visualize the isotropic conductivity, MREIT requires at least two independent injected currents because an infinite number of isotropic conductivity distributions can generate the same internal conductivity [11, 15]. However, biological tissues show anisotropy due to asymmetric cellular structures. A few studies for anisotropic conductivity image reconstructions based on the projected current density have been studied. Even though they reported the experimental data using multiple independent injection currents, but still have difficulty determining the other three components of the conductivity tensor [16, 17].

Diffusion-weighted imaging (DWI) is an imaging modality which measures the random Brownian motion of water molecules within a voxel of tissues such as brain white matter and skeletal muscle by applying the diffusion-sensitizing magnetic field gradient [18–21]. The statistical relationship between the mobility and the diffusivity was established by Einstein:

$$\begin{array}{c} \langle \Delta r^{2}\rangle =6D\Delta t \end{array}$$

where 〈Δ*r*^2^〉 is the average squared displacement of a particle over the time interval, Δ*t*, and *D* is the diffusion coefficient which incorporates temperature and media viscosity properties.

In this paper, we aim to investigate the electrical properties including the current density and the anisotropic conductivity tensor by the external current injection through attached electrodes during tDCS. Adopting the effective macroscopic anisotropic tensor modeled by a two-phase anisotropic medium, the electrical conductivity tensor, the mobility of water molecules, and the water diffusion tensor have been analyzed in terms of the intra- and extra-cellular transport coefficients [22]. Based on the model, the anisotropic conductivity tensor map has been investigated using an approximate linear relationship between the conductivity tensor and those of the water diffusion tensor [23–25].

The electrical conductivity in the extracellular space (ECS) can be decomposed into the concentration of ions and the mobility of charge carriers such as ions and charged molecules. Using the linear relationship between the conductivity tensor and those of the water diffusion tensor, the conductivity tensor is a product of scalar scale parameter and the diffusion tensor, where the scalar scale parameter mainly reflects the concentration of ECS ions. We propose an update scheme to reconstruct the scale parameter. By analyzing the update scheme and investigating how the updated conductivity converge to the true conductivity tensor, we provide more accurate insight into the current flow patterns and the conductivity characteristics.

Despite various applications in tDCS clinical and cognitive researches, the electrodes position on the head is sensitive to the internal current flow delivered to the brain [26]. As the potential applications of the proposed method for electrical properties (current density, conductivity, and electrical field), we can directly simulate the internal current flow for arbitrary electrodes configuration by solving the forward elliptic partial differential equation with the reconstructed internal conductivity distribution. The proposed method can design the electrodes configuration that is able to target specific areas of the brain with the reconstructed electrical properties.

Through an animal experiment, we verify that the proposed method can provide the electrical properties including the current density, ion-concentration information, and the conductivity tensor map due to tDCS.

## Materials and methods

### Measurement of magnetic flux density

The current injected into the brain through tDCS produces the internal current density distribution. The induced internal current density induces the magnetic flux density, **B** = (*B*_*x*_, *B*_*y*_, *B*_*z*_), by the Biot-Savart law. An MRI scanner allows to measure the *z*-component magnetic flux density by the injected current. A multi-echo in injected current non-linear encoding (ICNE) MREIT by currents from the end of RF pulse additionally accumulates the magnetic flux density *B*_*z*_ as a total phase offset. By comparing the *l*-th complex data *S*^*l*^ after injection current and *S*^0^ without injection current, the *z*-component of magnetic flux density distribution generated by the injected current, $B_{z}^{l}$, can be measured:

$$\begin{array}{cc} B_{z}^{l}\left(x,y\right)=\frac{1}{\gamma T_{E^{l}}}{\text{tan}}^{-1}\left(\frac{\alpha^{l}\left(x,y\right)}{\beta^{l}\left(x,y\right)}\right), & l=1,\cdots ,N_{E} \end{array}$$

where *γ* = 26.75 × 10^7^ rad/Ts is the gyromagnetic ratio of the hydrogen, *N*_*E*_ is the number of echoes, *T*_*E*^*l*^_ is the *l*-th echo time, *α*^*l*^ and *β*^*l*^ are the imaginary and real parts of *S*^*l*^ and *S*^0^, respectively [10]. Due to a small amount of injection current magnitude by tDCS (1-2 mA), the intensity of measured *B*_*z*_ data is within several nT ranges and the measured *B*_*z*_ data include non-negligible noise artifact. The weighted combination $B_{z}=\sum_{l=1}^{N_{E}}w_{l}B_{z}^{l}$ using the solved weighting factor *w*_*l*_ reduces noise artifacts of magnetic flux density [27]. The noise standard deviation of *B*_*z*_, *sd*(*B*_*z*_), depends on the intensity of MR signal and the time width of injection current:

$$\begin{array}{c} sd\left(B_{z}\right)\propto \frac{1}{T_{c}\left|S^{c}\right|} \end{array}$$

where *S*^*c*^ is the measured complex signal and *T*_*c*_ is the time width of injection current per repetition time (*T*_*R*_).

### Initial current density

Using the background conductivity *σ*_0_, we can generate an initial current density **J**^0^ = −*σ*_0_∇*u*^0^ by solving the following equation:

$$\begin{array}{c} \left\{ \begin{array}{cccc} \nabla \cdot \sigma_{0}\nabla u^{0} & = & 0\;\; & \text{in}\;\Omega \\ -\sigma_{0}\nabla u^{0}\cdot \nu & = & g\;\; & \text{on}\;\partial \Omega \end{array}\right. \end{array}$$

where *g* is the injected current density through the attached electrodes, *ν* is the outward normal vector, and Ω is an imaging object.

The initial current density **J**^0^ satisfies the divergence-free condition ∇ · **J**^0^ = 0. According to the Biot-Savart law

$$B_{z}^{0}\left(r\right)=\frac{\mu_{0}}{4\pi }\int \frac{\left(y-y\prime )J_{x}^{0}(r\prime )-(x-x\prime )J_{y}^{0}(r\prime \right)}{{\left|r-r\right|}^{3}}dr^{\prime },$$

the *z*-component of magnetic flux density can be easily updated from the current density.

### Relationship between conductivity tensor and diffusion tensor

Let **D** denote a 3 × 3 positive definite symmetric diffusion tensor matrix from DTI, which measures the intrinsic diffusion of water molecules in tissue:

$$\begin{array}{c} D=S_{D}\tilde{D}S_{D}^{T}\;\text{with}\;\tilde{D}=(\begin{array}{cccc} & \lambda_{d_{1}} & 0 & 0\\ & 0 & \lambda_{d_{2}} & 0\\ & 0 & 0 & \lambda_{d_{3}} \end{array}) \end{array}$$

where the column vectors of **S**_*D*_ = {**s**_1_, **s**_2_, **s**_3_} are the orthonormal eigenvectors of **D**, the superscript *T* denotes the transpose and *d*_*i*_ for *i* = 1, 2, 3 are the corresponding eigenvalues. The signal intensity *ρ*_*D*_ of diffusion MRI is given by

$$\begin{array}{c} \rho^{j}=\rho_{0}\text{exp}\left(-bg_{j}^{T}Dg_{j}\right),\;j=1,\cdots ,N_{d} \end{array}$$

where *ρ*_0_ is the signal obtained without diffusion-sensitizing gradient, **g**_*j*_ is the *j*-th normalized diffusion-sensitizing gradient vector and *b* denotes the diffusion-weighting factor depending on the gradient pulse used in the DT-MRI sequence. The *b*-value can be expressed as

$$\begin{array}{c} b=\gamma^{2}\delta^{2}G^{2}\left(\Delta -\frac{\delta }{3}\right) \end{array}$$

where *δ*, Δ, and *G* are the duration of applied gradient, the duration between the paired gradients, and amplitude, respectively.

By accepting the effective macroscopic anisotropic tensor model by Sen and Torquato [22], the effective electrical conductivity tensor in the case of macroscopically anisotropic media shares the eigenvectors with the water diffusion tensor allowing the well-known Hashin-Shtrikman bounds [28]. The eigenvalues $\lambda_{\sigma_{i}}$ of the conductivity tensor **C**^*σ*^ satisfy the following relation for the relatively small intra-cellular diffusion coefficient *d*_*int*_:

$$\begin{array}{ll} \lambda_{\sigma_{i}} & =\frac{\sigma_{ext}}{d_{ext}}\left[\lambda_{d_{i}}\left(\frac{d_{int}}{3d_{ext}}+1\right)+\frac{\lambda_{{}_{d_{i}}}^{2}d_{int}}{3d_{ext}^{2}}+\frac{2}{3}d_{int}\right]\\ & +O\left(d_{int}^{2}\right),\;i=1,2,3 \end{array}$$

where *σ*_*ext*_ is the extra-cellular conductivity, *d*_*ext*_ is the extra-cellular diffusion coefficient and $O(d_{int}^{2})$ is bounded as $d_{int}^{2}$ tends to infinity. Based on the effective anisotropic conductivity tensor model, the eigenvalues of the electrical conductivity and the water diffusion tensors approximately satisfy a linear relationship:

$$\begin{array}{c} C^{\sigma }=\eta S_{D}\tilde{D}S_{D}^{T}=\eta D \end{array}$$ (1)

where the coefficient *η* denotes a scale parameter between the eigenvalues $\lambda_{\sigma_{i}}$ and $\lambda_{d_{i}}$, *i* = 1, 2, 3. The scale parameter *η* is a scalar function influenced by the coefficients of *d*_*int*_, *d*_*ext*_, *σ*_*ext*_, and *d*_*ext*_. For the anisotropic conductivity tensor **C**^*σ*^, the divergence-free condition of current density and applied external current density satisfy

$$\begin{array}{c} \left\{ \begin{array}{cccc} \nabla \cdot J=-\nabla \cdot (C^{\sigma }\nabla u) & = & 0\;\; & \text{in}\;\Omega \\ J\cdot \nu =-C^{\sigma }\nabla u\cdot \nu & = & g\;\; & \text{on}\;\partial \Omega \end{array}\right. \end{array}$$ (2)

The relation (1) implies that

$$\begin{array}{c} \nabla \cdot \left(C^{\sigma }\nabla u\right)=\nabla \cdot (\eta S_{D}\tilde{D}S_{D}^{T}\nabla u)=0\;\text{in}\;\Omega \end{array}$$

### Computation of projected current density J^*p*^

Due to the limitation of the measured *B*_*z*_ signal which is the *z*-th component of magnetic flux density induced by tDCS, the vector field **J**^*p*^ as the projected current density of **J** is directly calculated from the measured *B*_*z*_ data as follows

$$\begin{array}{c} J^{p}\;≔\;J_{0}+{\tilde{\nabla }}^{⊥}\psi \;\;\text{in}\;\Omega_{t} \end{array}$$

where Ω_*t*_ is an imaging slice of Ω, **J**_0_ is the background current density, and the potential *ψ* solves

$$\{\begin{array}{cccc} {\tilde{\nabla }}^{2}\psi & = & \frac{1}{\mu_{0}}\nabla^{2}B_{z} & \text{in}\;\Omega_{t}\\ {\tilde{\nabla }}^{⊥}\psi \cdot \nu & = & 0 & \text{on}\;\partial \Omega_{t}. \end{array}$$ (3)

Here, we use the following two-dimensional terminologies:

$$\begin{array}{ll} \tilde{\nabla }f\;≔\;\left(\frac{\partial f}{\partial x},\frac{\partial f}{\partial y},0\right), & {\tilde{\nabla }}^{⊥}f\;≔\; \end{array}\left(\frac{\partial f}{\partial y},-\frac{\partial f}{\partial x},0\right).$$

The projected current density **J**^*p*^ is the quantity that is optimally observable from the measured *B*_*z*_ data [13]:

$${\left\|J-J^{p}\right\|}_{\Omega_{t}}\leq C\left({\left\|\frac{\partial }{\partial z}\left(J_{z}-J_{0,z}\right)\right\|}_{\Omega_{t}}+{\left\|J_{z}-J_{0,z}\right\|}_{\Omega_{t}}\right)$$

where ${\left\|\cdot \right\|}_{\Omega_{t}}$ denotes the *L*^2^-norm and the constant *C* does not depends on **J**.

### Diffusion tensor J-substitution algorithm

From the measured diffusion tensor and the recovered current density, the water diffusion coefficient can distinguish the discontinuous regions classified according to the diffusion difference of water molecules. The proposed DT-**J**-substitution algorithm is based on the distinguishable diffusivity signals of diffusion tensor across the discontinuous regions. Since a single-shot echo planar imaging (SS-EPI) pulse sequence is typically used to measure the diffusion tensor map, the measured each component of **D** is noisy due to the fast scan MR pulse sequence. The reconstructed current density **J**^*p*^ from the measured *B*_*z*_ data also contains non-negligible noise due to a small amount of injection current.

Utilizing the informations of **J**^*p*^(≈ **J**) and **D**, the aim is to determine the scale parameter that satisfies the following condition:

$$\begin{array}{c} \min_{\eta }∥\;\eta D\nabla u+J\;∥ \end{array}$$

Since the noise level of *B*_*z*_ is inversely proportional to the magnitude intensity, we introduce a neighborhood $N_{r}=\left\{r_{i}:i=1,\cdots ,N\right\}$ of a voxel **r** and set a weighting factor as

$$\begin{array}{c} w_{i}=e^{-h∥S\left(r_{i}\right)-S\left(r\right)∥}/\sum_{r_{j}\in N_{r}}e^{-h∥S\left(r_{j}\right)-S\left(r\right)∥} \end{array}$$

where *S* is the magnitude of the MR image and *h* is a function of the noise level of *B*_*z*_.

We propose an iteratively formula for the anisotropic conductivity tensor **C**^*σ*^:

$$\eta^{n+1}\left(r\right)=-\frac{\left(\sum_{r_{j}\in N_{r}}w_{j}J\left(r_{j}\right)\right)\cdot \left(\sum_{r_{j}\in N_{r}}w_{j}\nabla u^{n}\left(r\right)\right)}{\left(\sum_{r_{j}\in N_{r}}w_{j}D\left(r_{j}\right)\nabla u^{n}\left(r_{j}\right)\right)\cdot \left(\sum_{r_{j}\in N_{r}}w_{j}\nabla u^{n}\left(r_{j}\right)\right)}$$ (4)

The *n*-th updated voltage potential *u*^*n*^ satisfies

$$\begin{array}{c} \left\{ \begin{array}{ccc} \nabla \cdot (\eta^{n}D\nabla u^{n}) & =0 & \text{in}\;\Omega \\ \eta^{n}D\nabla u^{n}\cdot \nu & =g & \text{on}\;\partial \Omega \end{array}\right. \end{array}$$ (5)

Here, the voltage potential *u*^*n*^ is uniquely determined up to a constant.

The proposed algorithm for the current density **J**^*p*^, and the conductivity tensor **C**^*σ*^, using only tDCS can distinguish the discontinuous regions.

**Fig 1:**
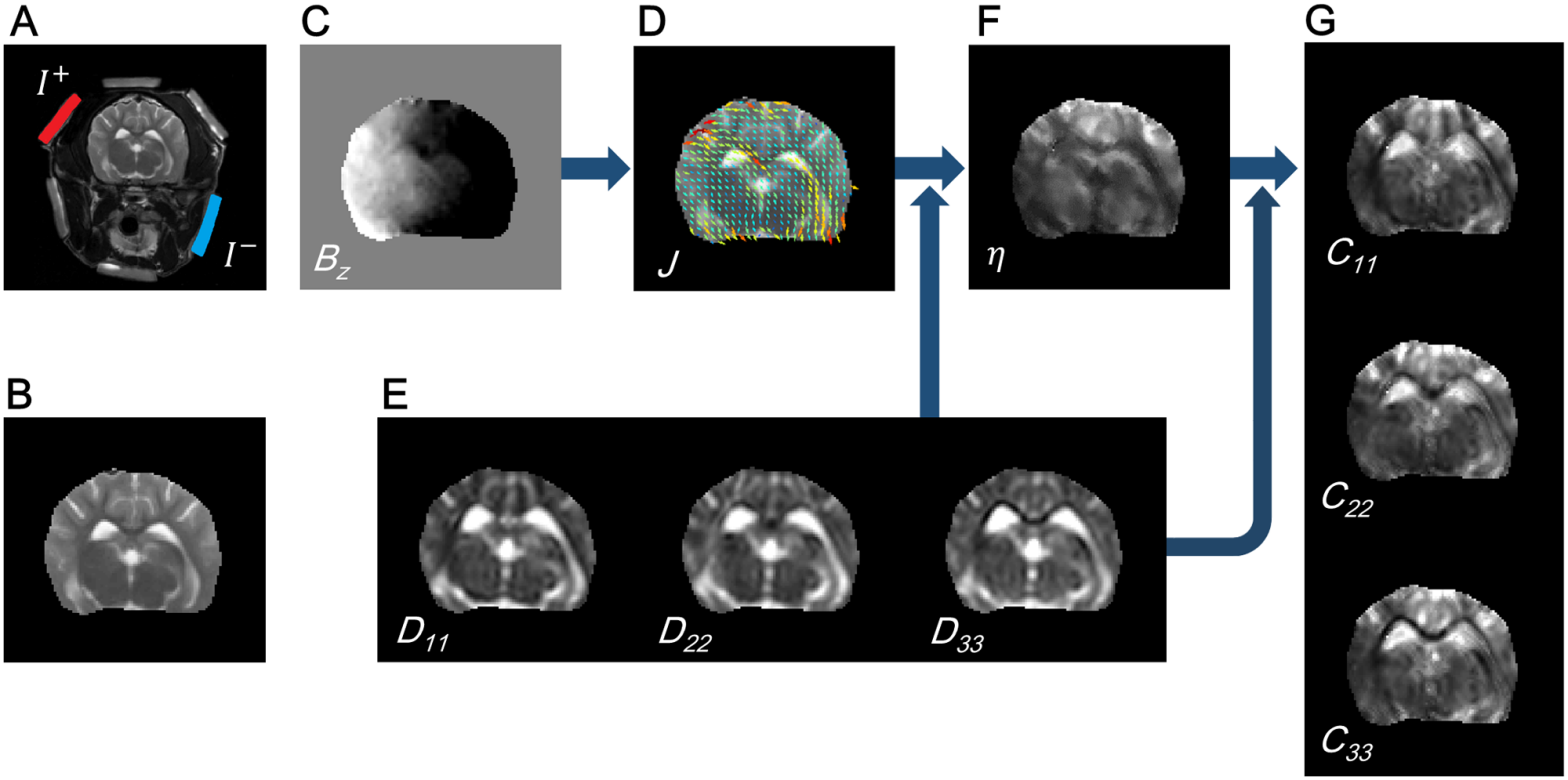
Schematic diagram for DT-J-substitution algorithm. (A) MR *T*_2_-weighted image of the canine head with three pairs of surface electrodes. A current is diagonally injected from the left-top to the right-bottom. (B) MR magnitude image of the brain region. (C) Measured *B*_*z*_ image for the current injection. (D) Recovered projected current flow overlaid on the MR magnitude image. (E) Diagonal components of the estimated diffusion tensor. (F) Recovered scale parameter to connect the conductivity tensor and the diffusion tensor. (G) Recovered electrical conductivity tensor.

Fig 1 shows a schematic diagram for the proposed DT-**J**-substitution algorithm:

1. Measure the *z*-component of magnetic flux density induced by tDCS current (Fig 1C).
2. Reconstruct the projected current **J**^*p*^ by solving the two dimensional Poisson’s equation in (3) (Fig 1D).
3. Reconstruct the scale parameter *η* in Fig 1F by the proposed DT-**J**-substitution scheme in (4).
4. Reconstruct the conductivity tensor by combining *η* and the diffusion tensor map.

### Characteristics of DT-J-substitution algorithm

For a discontinuous anomaly *D*_*σ*_ and a point *ξ* ∈ ∂*D*_*σ*_, the relation between the conductivity and the current flow satisfies

$$\begin{array}{cc} J^{+}\left(\xi \right)\cdot \nu \left(\xi \right) & ={\left(C^{\sigma }\right)}^{+}\left(\xi \right)\nabla u^{+}\left(\xi \right)\cdot \nu \left(\xi \right)\\ & ={\left(C^{\sigma }\right)}^{-}\left(\xi \right)\nabla u^{-}\left(\xi \right)\cdot \nu \left(\xi \right)=J^{-}\left(\xi \right)\cdot \nu \left(\xi \right) \end{array}$$ (6)

where *u*^+^ and *u*^−^ denote

$$\begin{array}{c} u^{+}=u{{|}_{{\bar{D}}_{\sigma }^{c}}\;\;\text{and}\;\;u^{-}=u|}_{D_{\sigma }}. \end{array}$$

The normal component of the current density **J** is continuous across the interface of the discontinuous anomaly. The relation (6) implies that only one current flow for visualizing discontinuous conductivity tensor **C**^*σ*^ is insufficient to distinguish the edge of anomaly.

Since the updated scale parameter *η*^*n*+1^ is determined by satisfying the condition, −*η*^*n*+1^**D**∇*u*^*n*^ ≈ **J** = −*η***D**∇*u*, we have the following identity:

$$\begin{array}{cc} \left({\left(\eta^{n+1}\right)}^{2}-\eta^{2}\right)\left(D\nabla u^{n}\right)\cdot \nabla u^{n} & =\left(\eta^{n+1}D\nabla u^{n}\right)\cdot \eta^{n+1}\nabla u^{n}-\eta^{2}\left(D\nabla u^{n}\right)\cdot \nabla u^{n}\\ & =J\cdot \left(D^{-1}J\right)-\eta^{2}\left(D\nabla u^{n}\right)\cdot \nabla u^{n}\\ & =\eta^{2}(\left(D\nabla u\right)\cdot \nabla u-\left(D\nabla u^{n}\right)\cdot \nabla u^{n}) \end{array}$$ (7)

Using the identity (7), the integration by part leads to

$$\begin{array}{l} \int_{\Omega }\frac{{\left(\eta^{n+1}\right)}^{2}-\eta^{2}}{\eta }\left(D\nabla u^{n}\right)\cdot \nabla u^{n}dr\\ \begin{array}{cc} & \;=\int_{\Omega }\eta \left(\left(D\nabla u\right)\cdot \nabla u-\left(D\nabla u^{n}\right)\cdot \nabla u^{n}\right)dr \end{array} \end{array}$$ (8)

The right hand side of (8) can be rewritten as

$$\begin{array}{l} \int_{\Omega }\eta (\left(D\nabla u\right)\cdot \nabla u-\left(D\nabla u^{n}\right)\cdot \nabla u^{n})dr\\ \begin{array}{cc} & \;=\int_{\Omega }\left(\eta^{n}-\eta \right)\left(D\nabla u^{n}\right)\cdot \left(\nabla u^{n}+\nabla u\right)dr \end{array}\\ \begin{array}{cc} & \;+\int_{\Omega }\left(\eta D\nabla u-\eta^{n}D\nabla u^{n}\right)\cdot \left(\nabla u^{n}+\nabla u\right)dr \end{array} \end{array}$$ (9)

The applied external injection current intensity *g* in (5) and the integration by part yield

$$\begin{array}{l} \int_{\Omega }\left(\eta D\nabla u-\eta^{n}D\nabla u^{n}\right)\cdot \left(\nabla u^{n}+\nabla u\right)dr\\ \begin{array}{cc} & \;=-\int_{\Omega }\nabla \cdot \left(\eta D\nabla u-\eta^{n}D\nabla u^{n}\right)\left(u^{n}+u\right)dr \end{array}\\ \begin{array}{cc} & \;+\int_{\partial \Omega }\left(\eta D\nabla u\cdot \nu -\eta^{n}D\nabla u^{n}\cdot \nu \right)\left(u^{n}+u\right)dS \end{array}\\ \begin{array}{cc} & \;=0 \end{array} \end{array}$$ (10)

By the relations (8), (9), and (10), we have the following identity

$$\begin{array}{l} \int_{\Omega }\left(\eta^{n+1}-\eta \right)\left(1+\frac{\eta^{n+1}}{\eta }\right)\left(D\nabla u^{n}\right)\cdot \nabla u^{n}dr\\ \begin{array}{cc} & \;=\int_{\Omega }\left(\eta^{n}-\eta \right)\left(D\nabla u^{n}\right)\cdot \left(\nabla u^{n}+\nabla u\right)dr \end{array} \end{array}$$ (11)

The identity (11) shows that the updated scheme is deeply related to the directions of **D**∇*u*^*n*^ and ∇*u*. From the measured diffusion tensor and the current density, for an anomaly region *D*_*σ*_ with different electrical concentrations of ions, we have the following relationship at a point *ξ* ∈ ∂*D*_*σ*_:

$$\begin{array}{cc} (D^{-1}\;J^{+}\left(\xi \right))\cdot \nu \left(\xi \right) & =\eta^{+}\nabla u^{+}\left(\xi \right)\cdot \nu \left(\xi \right)\\ & \neq \eta^{-}\nabla u^{-}\left(\xi \right)\cdot \nu \left(\xi \right)=\left(D^{-1}J^{-}\left(\xi \right)\right)\cdot \nu \left(\xi \right) \end{array}$$ (12)

The distinguishable signals of **D** on the edges of anomalies can updated the conductivity tensor even with the measured one component of magnetic flux density.

### Animal preparation

In this paper, we verify the proposed method with a canine brain experiment that used previously to visualize the conductivity tensor using two independent current injections and ADCs with 32 diffusion gradient directions [15]. We select a partial data set using one injection current and ADCs to estimate the diffusion tensor. A healthy laboratory beagle (3-4 years old; weighing 5-10 kg; Harlan Interfauna, Huntingdon, Cambridgeshire, UK) was used for imaging experiments. The dog had no signs of neurologic problems on physical examination. The dog was screened for metabolic disease by means of CBC and serum chemistry analysis and acclimated to the facility for a minimum of 1 week prior to being used in this study. The dog was housed individually and fed twice daily with a commercial dry food (Science Diet, Hill’s Pet Nutrition, Topeka, KS). Fresh water was supplied continuously by an automatic dispenser at our well-ventilated facility under controlled light-dark cycles (light on, 08:00 to 20:00). An indoor temperature range of 18 to 24°C and a humidity level of 55% ± 10% with 8 air changes hourly were maintained. Before MR imaging experiments, we injected 0.1 mg/kg of atrophine sulfate to prevent dribbling. Ten minutes later, we anesthetized the dogs with an intramuscular injection of 0.2 ml/kg Zolazepam (Zoletil 50, Virbac, France). After clipping and shaving the hair around the chosen imaging area, we attached three pairs of carbon-hydrogel (HUREV Co. Ltd, Korea) surface electrodes (the size of each electrode was 30 × 30 × 5 mm^3^) to the skin. During MR scanning, we intubated the animal using an endotracheal tube of 8.5 mm diameter and began general anesthesia using an anesthesia system (VME, MATRX, USA). We used 2% isoflurane mixed with oxygen at 800 ml/min flow rate. Ventilation was machine-controlled using a mechanical ventilator (M-2002, Hallowell EMC, USA) with 15 bpm respiration rate and 200 ml tidal volume. The animal experiments were approved by the Kyung Hee University Institutional Animal Care and Use Committee (Permit number: KHUASP(SE)-14-25) and performed under the guidelines of the Committee.

## Results

We injected 2 mA currents in the diagonal direction, from the left top to the right bottom in Fig 1A, using a custom-designed MREIT current source and acquired MR images using the coherent steady state multi-gradient echo (CSS-MGRE) pulse sequence. After MREIT scans, we performed DT-MRI scans using the single-shot spin-echo echo planar imaging (SS-SE-EPI) pulse sequence to measure the diffusion coefficient for the three diffusion gradient directions. Table 1 shows the parameters for the MR experiments.

**Table 1:** Parameters for imaging experiments.

	Image size	FOV(mm^3^)	TR/TE(ms)	*N*_*E*_/NEX
MREIT	128 × 128 × 6	160 × 160 × 30	300/2.3	13/35
DWI	112 × 112 × 6	160 × 160 × 30	8000/94	1/2
	*b*-value = 800 s/mm^2^, 32 diffusion gradient directions			

**Fig 2:**
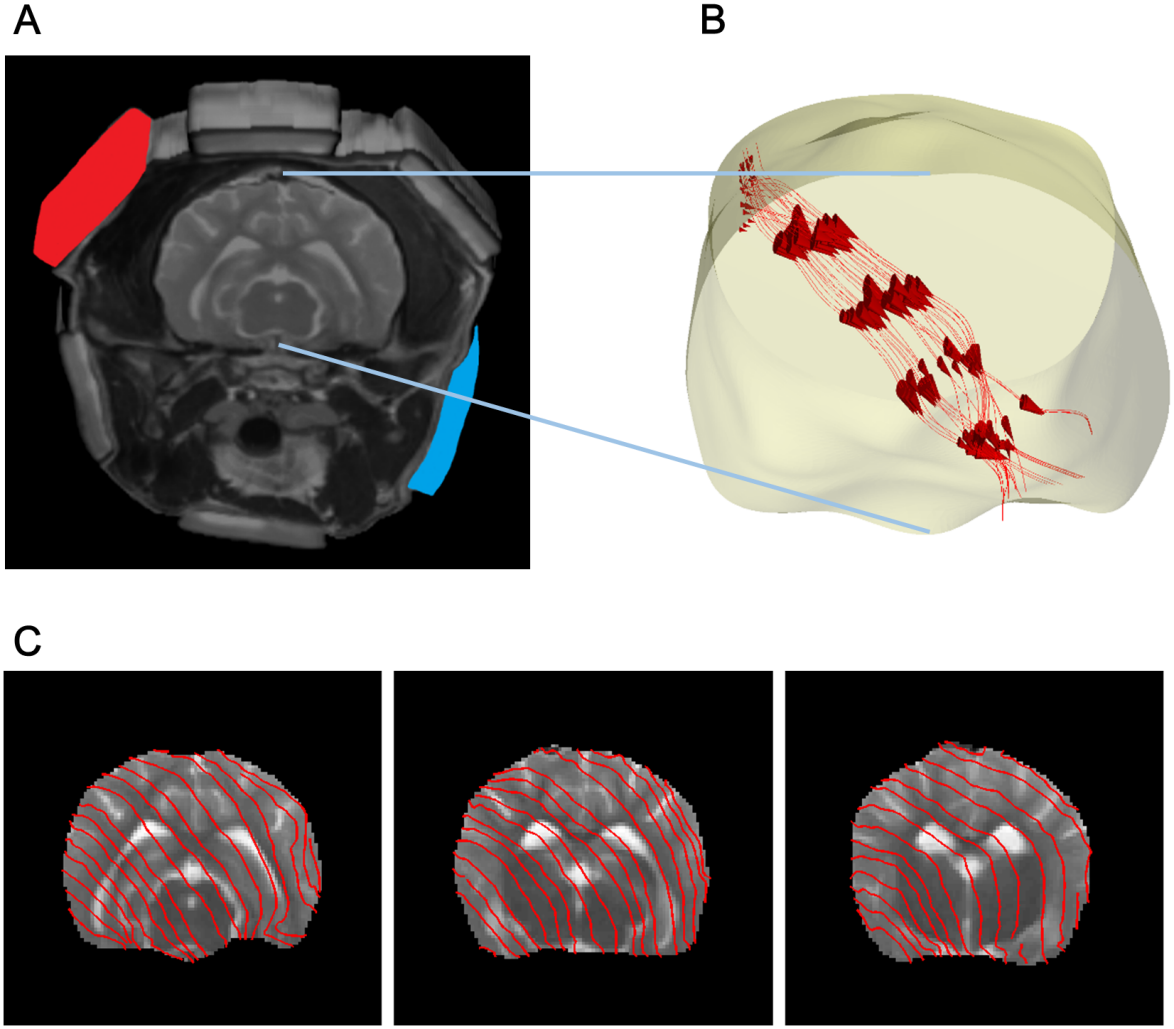
Internal current density in the brain region by injecting current. (A) Cylindrical imaging structure attached a pair of electrodes to inject current. (B) Three dimensional current flow in the brain region. (C) Current flow streamlines overlap the *T*_2_ weighted MR magnitude image in the first, second, and third slices.

Fig 2A shows the cylindrical structure by accumulating the imaging slices. Fig 2B shows the three dimensional current flow in the brain region. In Fig 2C, the recovered projected current densities were displayed by the current flow streamlines overlap the *T*_2_ weighted image in the first, second, and third slices. See S1 Fig for the three dimensional current flow in the brain region by injecting current through another pair of attached electrodes.

**Fig 3:**
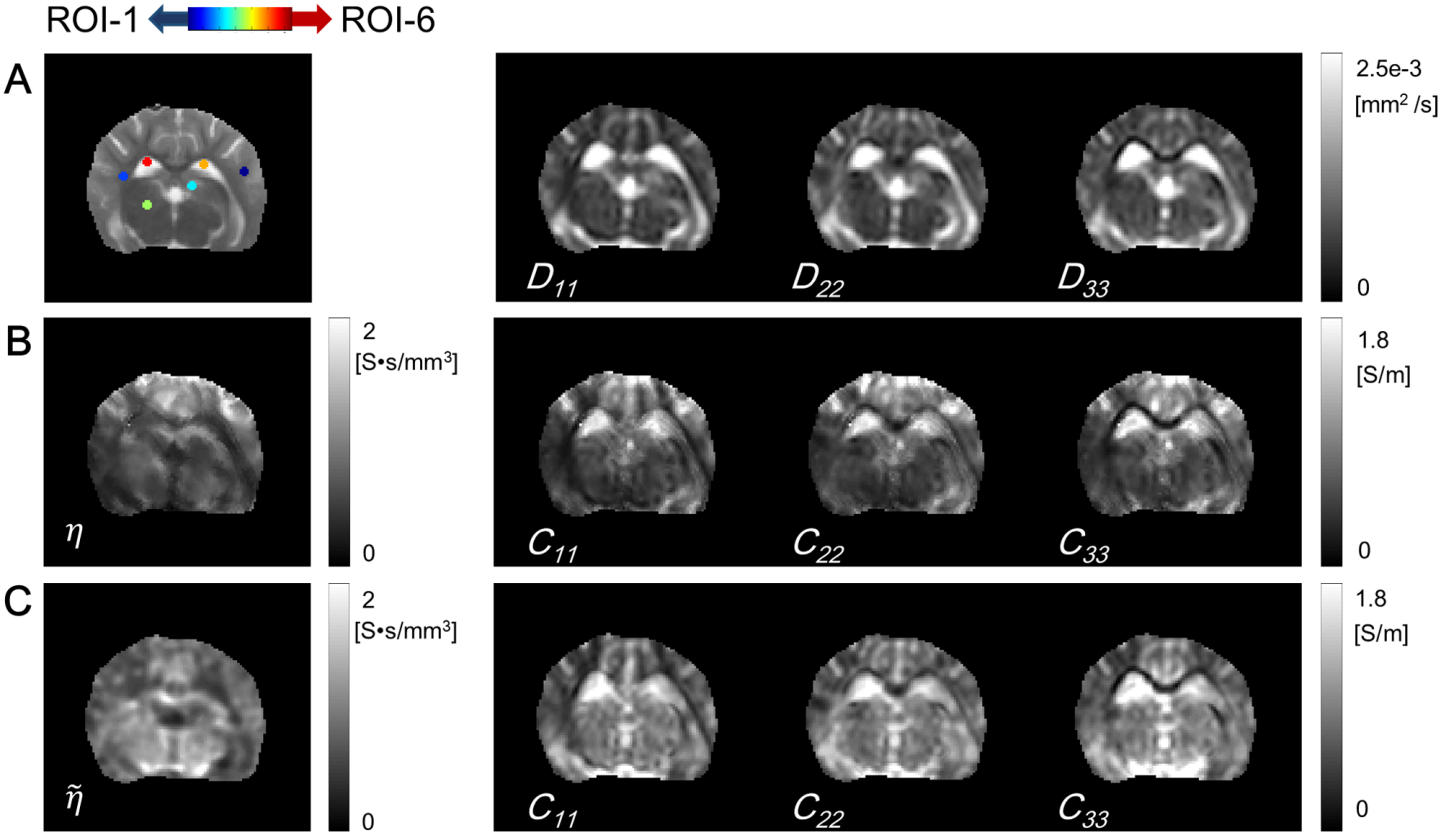
Diffusion tensor images and recovered electrical property images. (A) Six ROIs were overlaid on the *T*_2_ image and diagonal components of the diffusion tensor of water molecule. (B) Case 1: reconstructed scale parameter *η* and diagonal components of the conductivity tensor using tDCS current. (C) Case 2: reconstructed scale parameter $\tilde{\eta }$ and diagonal components of the conductivity tensor using two independent injection currents.

Fig 3A shows the estimated diagonal components of the diffusion tensor of water molecule, *D*_*ii*_, *i* = 1, 2, 3 and six regions of interest (ROIs) including a local region of 3 × 3 voxels to estimate the numerical values of the scale parameter *η* and the conductivity tensor. The six ROIs were selected with respect to the tissue anisotropy characteristics (white matter (ROI-1, 2), gray matter (ROI-3, 4), and CSF (ROI-5, 6)). Fig 3B shows the reconstructed scale parameter and the diagonal components of the conductivity tensor, **C**^*σ*^, using tDCS current. We updated 2-times by following (4). To suppress the noise artifact accumulated in the diffusion tensor and the current density, we chose a search neighborhood $N_{r}=\left\{r_{i}:i=1,\cdots ,25\right\}$ around an imaging voxel **r**. Since the noise artifact is severe in the measured *B*_*z*_ data, the range of *B*_*z*_ is within several nT, the weighting factor was determined in the $N_{r}$ as

$$\begin{array}{c} w_{i}=e^{-h∥S\left(r_{i}\right)-S\left(r\right)∥}/\sum_{j=1}^{25}e^{-h∥S\left(r_{j}\right)-S\left(r\right)∥} \end{array}$$

where *S* was the magnitude of the MR image and the denominator parameter *h* was 0.1. For the another pair of electrodes in S1 Fig, we recovered the scale parameter *η* and diagonal components of the conductivity tensor using the injected current (See S2 Fig).

In Fig 3C, we compared the recovered scale parameter $\tilde{\eta }$ using tDCS current with those using two independent injection currents. By substituting the linear relationship $C^{\sigma }=\tilde{\eta }D$ and utilizing the curl-free condition, ∇ × ∇*u* = 0, we have the following direct relation between the measured current density and diffusion tensor and the scale parameter:

$$\begin{array}{c} \nabla \text{ln}\;\tilde{\eta }\times \left(D^{-1}J^{p}\right)=\nabla \times \left(D^{-1}J^{p}\right). \end{array}$$

We directly recovered the scale parameter $\tilde{\eta }$ using two linearly independent injections currents by solving the following matrix system [25]:

$$\begin{array}{c} \begin{array}{c} \left(\begin{array}{cc} {\left(D^{-1}J^{p}\right)}_{y} & -{\left(D^{-1}J^{p}\right)}_{x}\\ {\left(D^{-1}J_{2}^{p}\right)}_{y} & -{\left(D^{-1}J_{2}^{p}\right)}_{x} \end{array}\right)\left(\begin{array}{c} \frac{\partial \;\text{ln}\;\tilde{\eta }}{\partial x}\\ \frac{\partial \;\text{ln}\;\tilde{\eta }}{\partial y} \end{array}\right)=\left(\begin{array}{c} \frac{\partial {\left(D^{-1}J^{p}\right)}_{x}}{\partial y}-\frac{\partial {\left(D^{-1}J^{p}\right)}_{y}}{\partial x}\\ \frac{\partial {\left(D^{-1}J_{2}^{p}\right)}_{x}}{\partial y}-\frac{\partial {\left(D^{-1}J_{2}^{p}\right)}_{y}}{\partial x} \end{array}\right) \end{array} \end{array}$$ (13)

where $J_{2}^{p}$ denotes the projected current density using the measured *B*_*z*_ data by injecting assistant injection current and (**D**^−1^**J**^*p*^)_*x*_ and (**D**^−1^**J**^*p*^)_*y*_ denote the *x*- and *y*-component of the vector **D**^−1^**J**^*p*^, respectively. In the second columns of Fig 3B and 3C, we compared the diagonal components of the reconstructed conductivity tensor using tDCS current and two independent injection currents, respectively. Table 2 shows the estimated values of the reconstructed scale parameters and diagonal components of diffusion tensors within the marked six ROIs in Fig 3A.

**Table 2:** The reconstructed *η* and the diagonal components of the conductivity tensor measured within the ROIs in Fig 3A.

		ROI-1	ROI-2	ROI-3	ROI-4	ROI-5	ROI-6
Case 1	*η*	0.78±0.06	0.77±0.16	0.53±0.07	0.73±0.04	1.06±0.14	0.62±0.04
	*C*_11_	0.51±0.09	0.41±0.14	0.65±0.08	0.55±0.03	1.62±0.18	1.55±0.12
	*C*_22_	0.55±0.03	0.66±0.12	0.74±0.08	0.46±0.04	1.52±0.21	1.54±0.13
	*C*_33_	0.79±0.05	0.42±0.15	0.70±0.12	0.51±0.03	1.64±0.18	1.64±0.14
Case 2	$\tilde{\eta }$	0.95±0.03	1.23±0.08	0.68±0.05	1.15±0.03	1.03±0.15	0.85±0.12
	*C*_11_	0.62±0.08	0.64±0.15	0.84±0.11	0.86±0.03	1.57±0.15	2.10±0.15
	*C*_22_	0.68±0.05	1.06±0.04	0.95±0.09	0.73±0.05	1.47±0.18	2.09±0.15
	*C*_33_	0.96±0.06	0.65±0.12	0.90±0.14	0.81±0.04	1.59±0.15	2.22±0.18

The proposed DT-**J**-substitution algorithm depends on a local behavior of the current density and the diffusion tensor from ADCs of water molecules. The reconstructed scale parameter using tDCS current in Fig 3B was influenced by the relationship between the current flow and the iteratively updated electrical field ∇*u*^*n*^.

**Fig 4:**
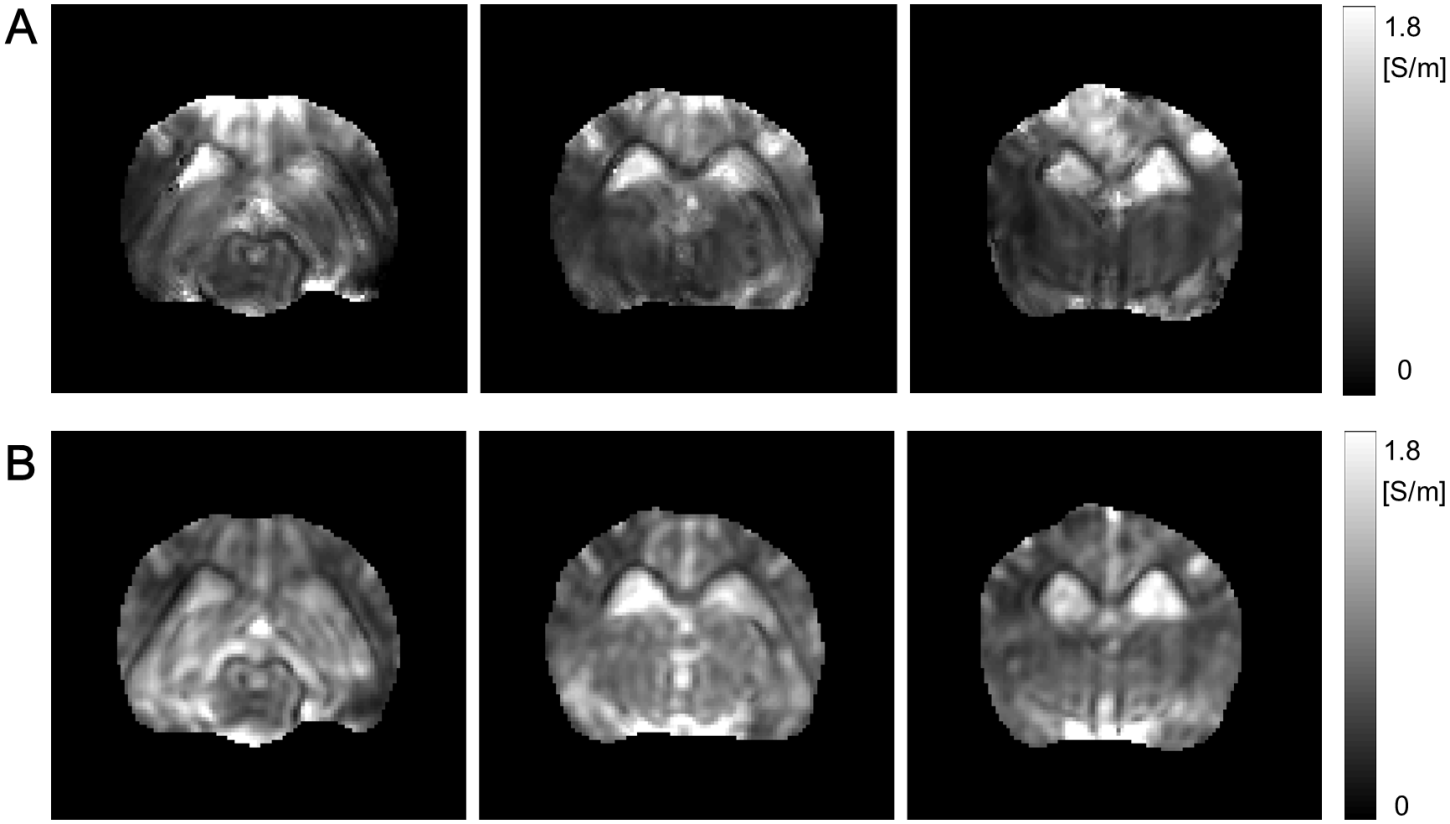
Mean conductivity images in the first, second, and third imaging slices. (A) Reconstructed mean conductivity images using tDCS current in the first, second, and third imaging slices. (B) Reconstructed mean conductivity images using two independent injection currents in the first, second, and third imaging slices.

Fig 4A shows the mean conductivity $\hat{c}=\frac{C_{11}^{\sigma }+C_{22}^{\sigma }+C_{33}^{\sigma }}{3}$ images using tDCS injection current in the first, second, and third imaging slices (See S3 Fig for the mean conductivity images using the another pair of attached electrodes). Fig 4B shows the corresponding images to Fig 4A, ${\hat{c}}_{2}$, using two injection currents. Since the current density $J_{2}^{p}$ compensated the weak region where the intensity of **J**^*p*^(**r**) · ∇*u*^*n*^(**r**) was small, the recovered mean conductivity using the two injection currents by combining the current densities **J**^*p*^ and the assistant current density $J_{2}^{p}$ was definitely advantageous comparing to that using tDCS current. However, the usage of two independent injection currents is not suitable for actual tDCS situations. In that sense, the reconstructed conductivity images in Fig 4A show the feasibility of the DT-**J**-substitution method.

The proposed DT-**J**-substitution algorithm iteratively updates the scale parameter *η* based on the identity (11). The identity (11) implies that the convergence of the iteratively updated *η*^*n*^ mainly relates to the angle between the velocity vector **D**∇*u*^*n*^ and the electric field **E** = −∇*u*:

$$\alpha ={\text{cos}}^{-1}\left(\frac{(D\nabla u^{n})\cdot \nabla u}{∥D\nabla u^{n}∥∥\nabla u∥}\right)$$ (14)

**Fig 5:**
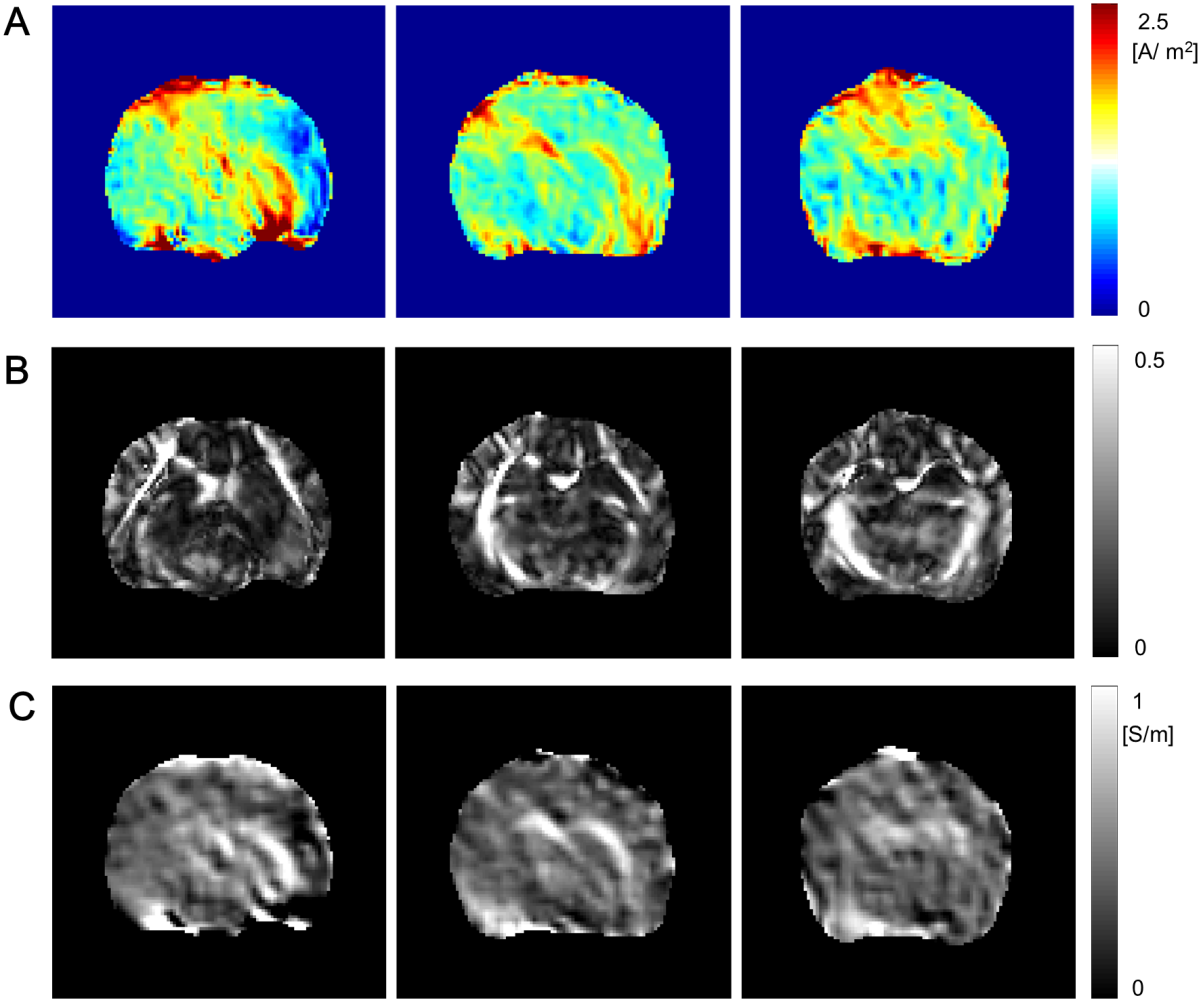
Recovered electrical property images. (A) Intensity of the projected current density, (B) Angle images between the twice updated velocity vector, **D**∇*u*^2^, and the electric field ∇*u*, and (C) Reconstructed apparent isotropic conductivity images by using **J**-substitution algorithm.

For the electric field **E**, we solved the forward problem (2) with the scale parameter $\tilde{\eta }$ in (13). Fig 5A and 5B show the intensity of the projected current density and the angle map *α* in (14), respectively. Comparing to the recovered mean conductivity images in Fig 4A, the contrast of the reconstructed mean conductivity relates to the characteristics of the current density and the angle map between the velocity vector and the electric field distribution. The DT-**J**-substitution algorithm uses the mismatch condition on the boundary of discontinuous region in (12). Without the diffusion tensor information, Fig 5C shows the apparent isotropic conductivity by using **J**-substitution algorithm using tDCS current:

$$\sigma^{n+1}\left(r\right)=-\frac{J^{p}\left(r\right)\cdot \nabla u^{n}\left(r\right)}{\nabla u^{n}\left(r\right)\cdot \nabla u^{n}\left(r\right)}$$

where the *n*-th updated voltage potential *u*^*n*^ satisfies

$$\begin{array}{c} \left\{ \begin{array}{ccc} \nabla \cdot (\sigma^{n}\nabla u^{n}) & =0 & \text{in}\;\Omega \\ \sigma^{n}\nabla u^{n}\cdot \nu & =g & \text{on}\;\partial \Omega \end{array}\right. \end{array}$$

Due to the relationship between the conductivity and the current flow in (6), the apparent isotropic conductivity in Fig 5C provided no distinguishable conductivity information in areas where the current flow was parallel to the electric field. By injecting current through the another pair of attached electrodes, see S4 Fig for the corresponding electrical property images in Fig 5.

**Fig 6:**
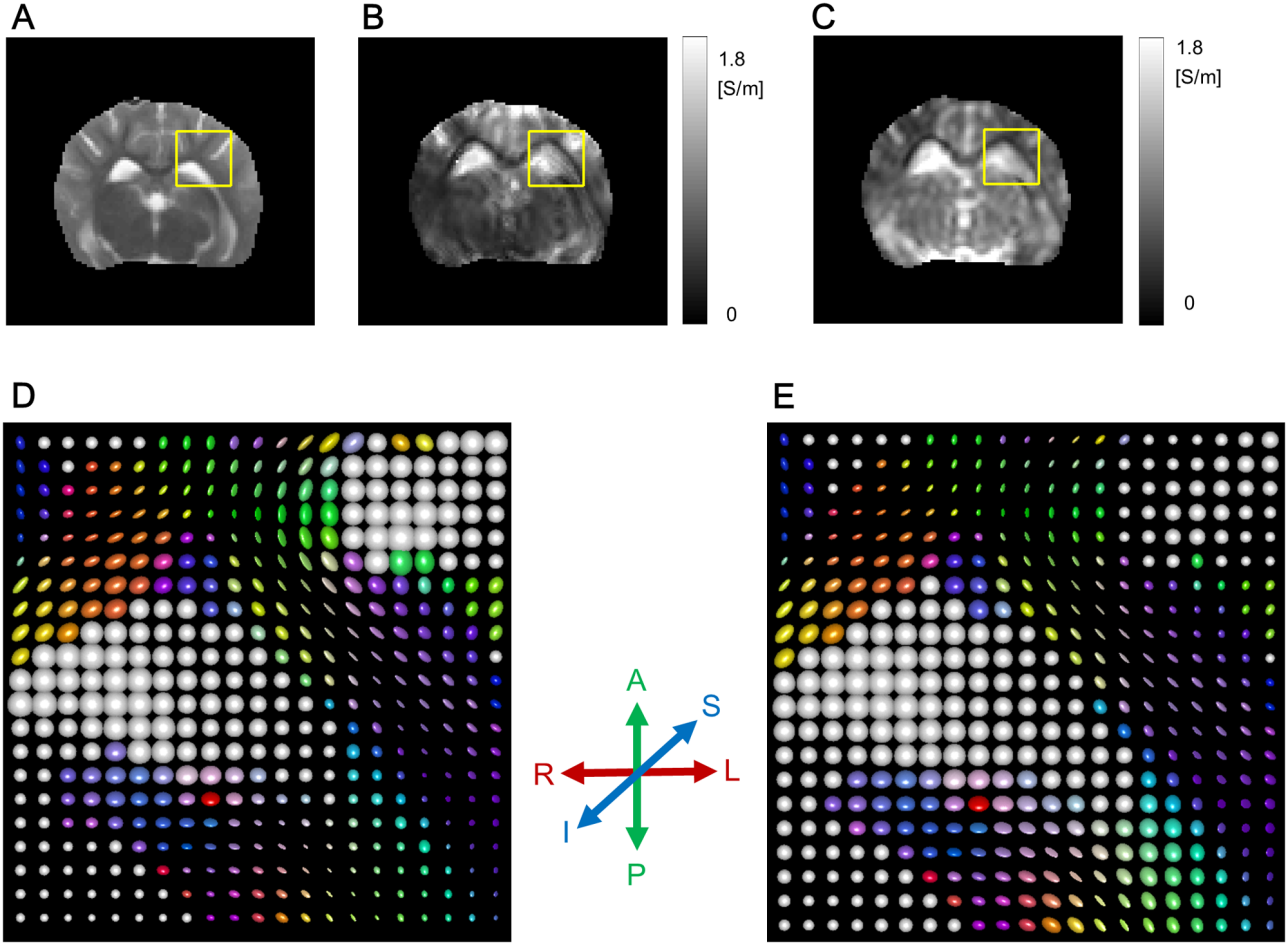
Conductivity tensors inside the chosen rectangular ROI. (A) Rectangular ROI marked in the *T*_2_-weighted MR magnitude image. (B) and (C) Mean conductivities reconstructed using tDCS current and two injection currents, respectively. (D) and (E) Conductivity tensor images corresponding to (B) and (C) represented by tri-axial ellipsoids, respectively. The radii of each ellipsoid are proportional to the eigenvalues and their axes are oriented along the directions of the eigenvectors. The colors of the ellipsoid indicate the orientation of the principle eigenvector.

Fig 6D and 6E compared the reconstructed conductivity tensor maps using tDCS current and two injection currents, respectively, inside the rectangular ROI shown in Fig 6A. Since both of the conductivity tensors shared the same eigenvectors from the diffusion tensor, their orientations were same. The position-dependent conductivity tensor image recovered by the proposed DT-**J**-substitution method were quite similar to the reconstructed conductivity tensor using the two injection currents. The elliptic radii in Fig 6D and 6E were proportional to the eigenvalues of the tensor at each voxel.

## Discussion

The measured magnetic flux density induced by tDCS dominantly reflects the extracellular effects because the cell membrane is regarded as an electrical insulator. The recovered current density directly from the measured *B*_*z*_ data also reflects only the ECS effects. However, The diffusion tensor of water molecules experiences other complex different environments even within a single voxel and includes the diffusion properties of intra- and extra-cellular compartments. The proposed method actually recovered the scalar scale parameter that connects the conductivity tensor and the diffusion tensor. The reconstructed conductivity tensor using the measured magnetic flux density induced by tDCS current and the diffusion tensor is influenced by the intra- and extra-cellular compartments due to the measured ADCs. In this paper, we assume that ADC at relatively low *b*-value(< 1000 s/mm^2^) dominates the fast diffusion component [29].

Based on the fact that the fast diffusion is proportional to extracellular diffusion, we used the diffusion tensor from ADCs with multiple diffusion gradient directions as an approximated extracellular diffusion tensor. In the proposed DT-**J**-substitution algorithm, the updated scale parameter satisfies the following structure:

$$\begin{array}{c} \eta^{n+1}D\nabla u^{n}=\eta^{n+1}\left(\alpha D_{E}+\left(1-\alpha \right)D_{I}\right)\nabla u^{n}=-J=-\eta \alpha D_{E}\nabla u \end{array}$$

where *α* is the volume fraction of ECS and **D**_*I*_ denotes the intracellular component of the diffusion tensor. By assuming a relatively small intracellular diffusion coefficient, the updated scale parameter contains some error by the intracellular volume fraction and **D**_*I*_. Using multi-*b* values and multi-diffusion gradient directions, the high angular resolution diffusion imaging (HARDI) technique has been introduced to overcome the limitation of DTI [23]. In recent years, non-Gaussian diffusion methods permit the analysis of the DW signal over a various range of *b*-values. The neurite orientation dispersion and density imaging (NODDI) requires at least two HARDI shells with different *b*-value for estimating the neurite morphology, the technique requires relatively long acquisition times [30]. The extracellular diffusion coefficient can be approximately estimated as a parameter using the NODDI technique. The diffusion models have been developed by considering the complexity and heterogeneity of the neuronal tissue microstructures. The proposed anisotropic conductivity model can be a different form depending on the diffusion model.

Although some specific issues (cables and electrodes, MR pulse sequence) need to be addressed when using typical tDCS device combining with the MR scanner, using the advent of MR-compatible tDCS systems, both sequential and concurrent acquisitions are possible using the conventional MR pulse sequences. In this paper, we used the coherent steady state multi-gradient echo (CSS-MGRE) MR pulse sequence to accumulate the phase signal by the injected current through tDCS and the single-shot spin-echo echo planar imaging (SS-SE-EPI) pulse sequence to measure the diffusion coefficient for the three diffusion gradient directions. For further issues for the blood-oxygen-level dependent (BOLD) contrast functional MRI and MR spectroscopy (MRS), MR field maps with and without tDCS should be carefully designed and analyzed. Combining with the electrical properties (conductivity, current density, and electric field), the interpretation of BOLD fMRI and MRS data has sufficient potential for future tDCS applications.

As a non-invasive brain imaging, fMRI is a popular method to the treatment of patients with neurological and neuropsycohological disorders, based on the increase in blood flow to the local area. The fMRI does not directly measure electrical activity since the blood flow is an indirect measure of neural activity. However, the reconstructed electrical properties using the proposed DT-**J**-substitution algorithm directly reflect the neural activity. Although complex neural activities may cause loss of signal due to self-cancellation of neural currents in an MR imaging voxel and the measured magnetic flux density signal is very sensitive to noise artifact, the proposed approach has potential to monitor neural electrical activity generated by the brain at a higher spatial resolution.

Transcranial alternating current stimulation (tACS) is a neuromodulatory technique similar to tDCS, which uses a sinusoidal AC current for noninvasive investigation of brain oscillations. tDCS effects depend on the applied frequency, amplitude, and phase. tACS applied currents to the scalp in the EEG frequency range (0.1-80Hz) can interfere with oscillatory brain activity. Although the tACS effects have been reported to involve manipulation and delivery of brain vibrations and corresponding biochemical changes, the exact mechanisms are still unclear. For the alternating current frequency for tACS, we can modify the MR pulse sequence instead of the CSS-MRGE which is appropriate for the injection current procedure of tDCS. To accumulate the magnetic flus density by the oscillatory current flow, we can design the MR pulse sequence by applying synchronized 180° rephasing pulses based on the multi-echo spin echo (ME-SE) pulse sequence [27].

We believe that the DT-**J**-substitution algorithm is a promising approach to obtain electrical properties in the brain by tDCS current. The proposed method reconstructs the electrical properties in a voxel-by-voxel by observing local behaviors of the current density and the diffusion tensor. Our future work will include the development of an efficient and stable algorithm for electrical property imaging by accounting for local and global characteristics of the current density and the diffusion tensor.

## Conclusion

We introduce a method to visualize the electrical properties including the electrical anisotropic conductivity, effective extracellular ion-concentration, and electric field using tDCS currents *in-vivo* by measuring ADCs and the magnetic flux density induced by tDCS. The proposed DT-**J**-substitution algorithm iteratively updates the scale parameter, which connects the anisotropic conductivity tensor to the diffusion tensor of water molecules. We analyze the relationship between the current flow and the scale parameter. We found that the contrast of reconstructed conductivity tensor by tDCS current mainly depended on the intensity of the current density and the angle between the velocity vector and the electric field. Through an animal experiment, we demonstrate the feasibility of the recovered electrical properties using a single tDCS current.

## Supporting information

S1 Fig Internal current density in the brain region by injecting current from the right top to the left bottom. (A) Cylindrical imaging structure attached a pair of electrodes to inject current. (B) Three dimensional current flow in the brain region. (C) Current flow streamlines overlap the *T*_2_ weighted MR magnitude image in the first, second, and third slices.
(TIF)

S2 FigReconstructed electrical properties.(A) Reconstructed scale parameter *η* and diagonal components of the conductivity tensor using tDCS current. (B) Reconstructed scale parameter $\tilde{\eta }$ and diagonal components of the conductivity tensor using two independent injection currents.(TIF)

S3 Fig Mean conductivity using the another pair of attached electrodes. (A) Reconstructed mean conductivity images using tDCS current in the first, second, and third imaging slices. (B) Reconstructed mean conductivity images using two independent injection currents in the first, second, and third imaging slices.
(TIF)

S4 Fig Recovered electrical property images in the first, second, and third imaging slices. (A) Intensity of the projected current density. (B) Angle images between the second updated velocity vector, **D**∇*u*^2^, and the electric field ∇*u*. (C) Reconstructed apparent isotropic conductivity images by using **J**-substitution algorithm.
(TIF)

S1 File ARRIVE checklist. This is the ARRIVE guidelines checklist for animal research reporting in vivo experiments.
(PDF)
