# Supplementary figures and images for: Anisotropic conductivity tensor imaging for transcranial direct current stimulation (tDCS) using magnetic resonance diffusion tensor imaging (MR-DTI)
Source: PLoS One. 2018 May 15;13(5):e0197063. doi: 10.1371/journal.pone.0197063 (PMC5953498; doi:10.1371/journal.pone.0197063)

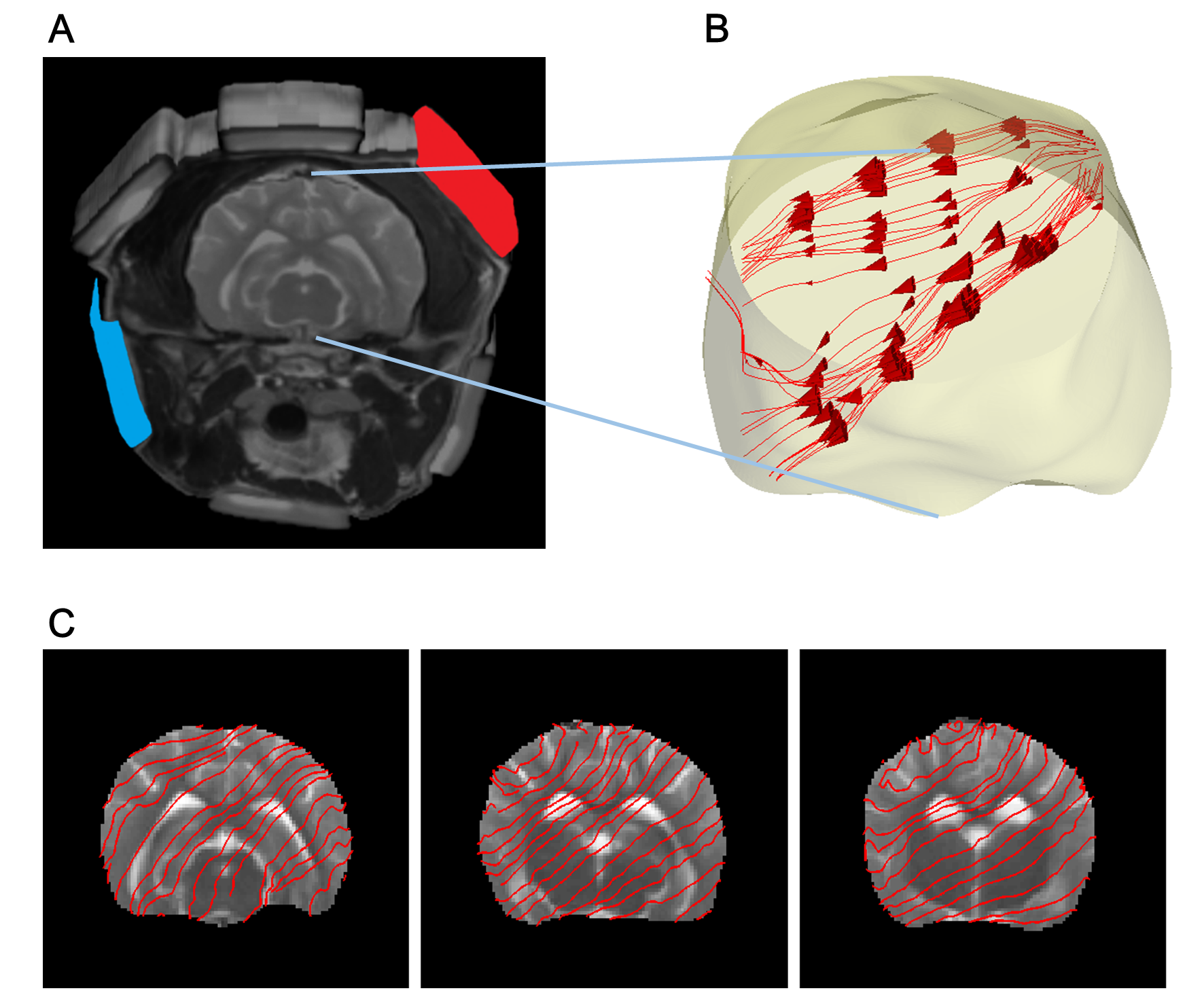

Supplement: S1 Fig — (A) Cylindrical imaging structure attached a pair of electrodes to inject current. (B) Three dimensional current flow in the brain region. (C) Current flow streamlines overlap the T2 weighted MR magnitude image in the first, second, and third slices. (TIF) [file pone.0197063.s001.tif]

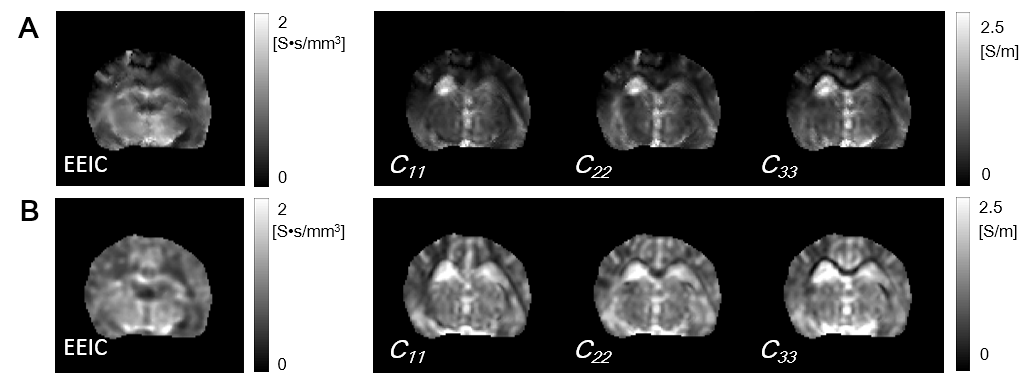

Supplement: S2 Fig — (A) Reconstructed scale parameter η and diagonal components of the conductivity tensor using tDCS current. (B) Reconstructed scale parameter η˜ and diagonal components of the conductivity tensor using two independent injection currents. (TIF) [file pone.0197063.s002.tif]

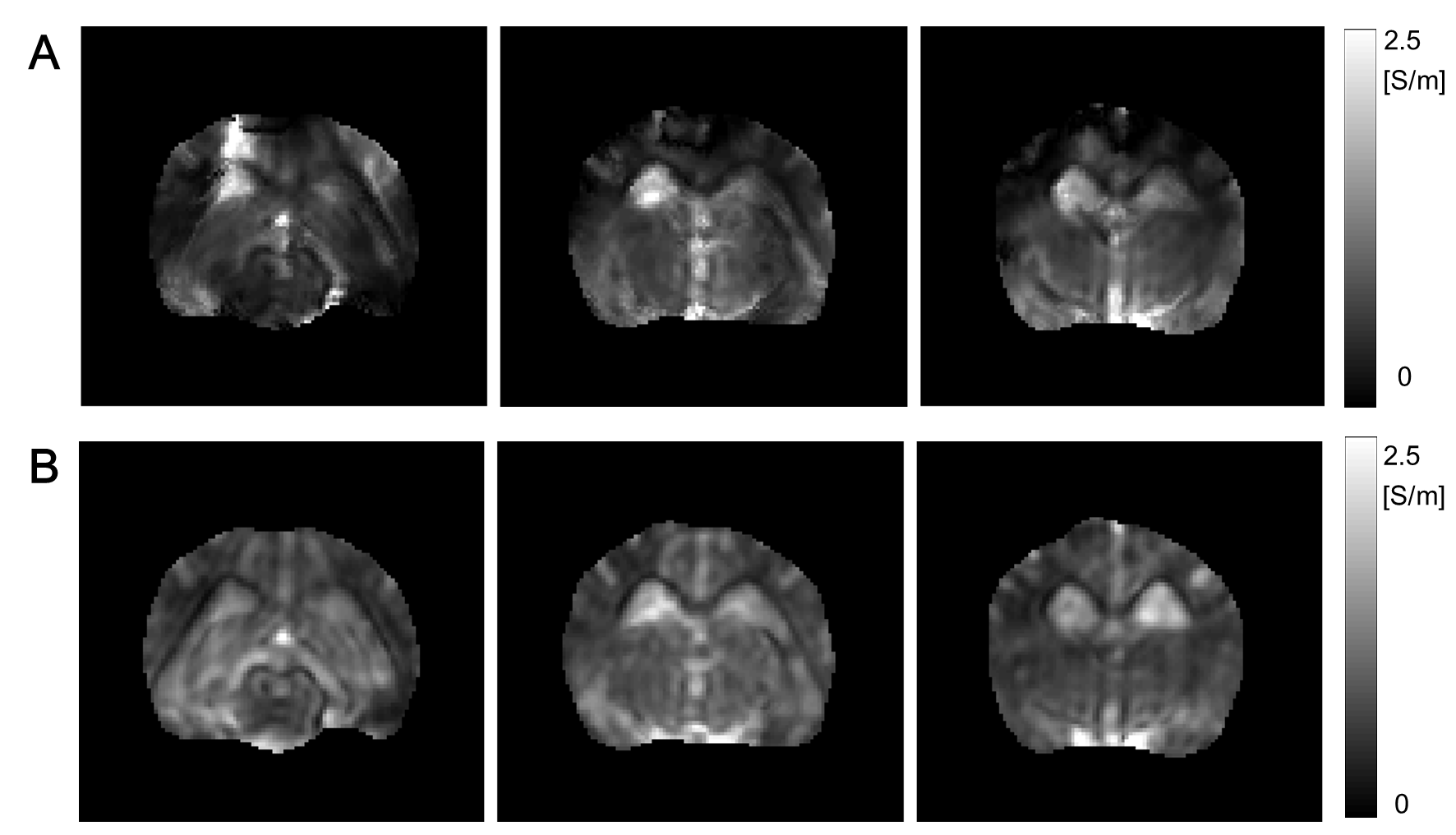

Supplement: S3 Fig — (A) Reconstructed mean conductivity images using tDCS current in the first, second, and third imaging slices. (B) Reconstructed mean conductivity images using two independent injection currents in the first, second, and third imaging slices. (TIF) [file pone.0197063.s003.tif]

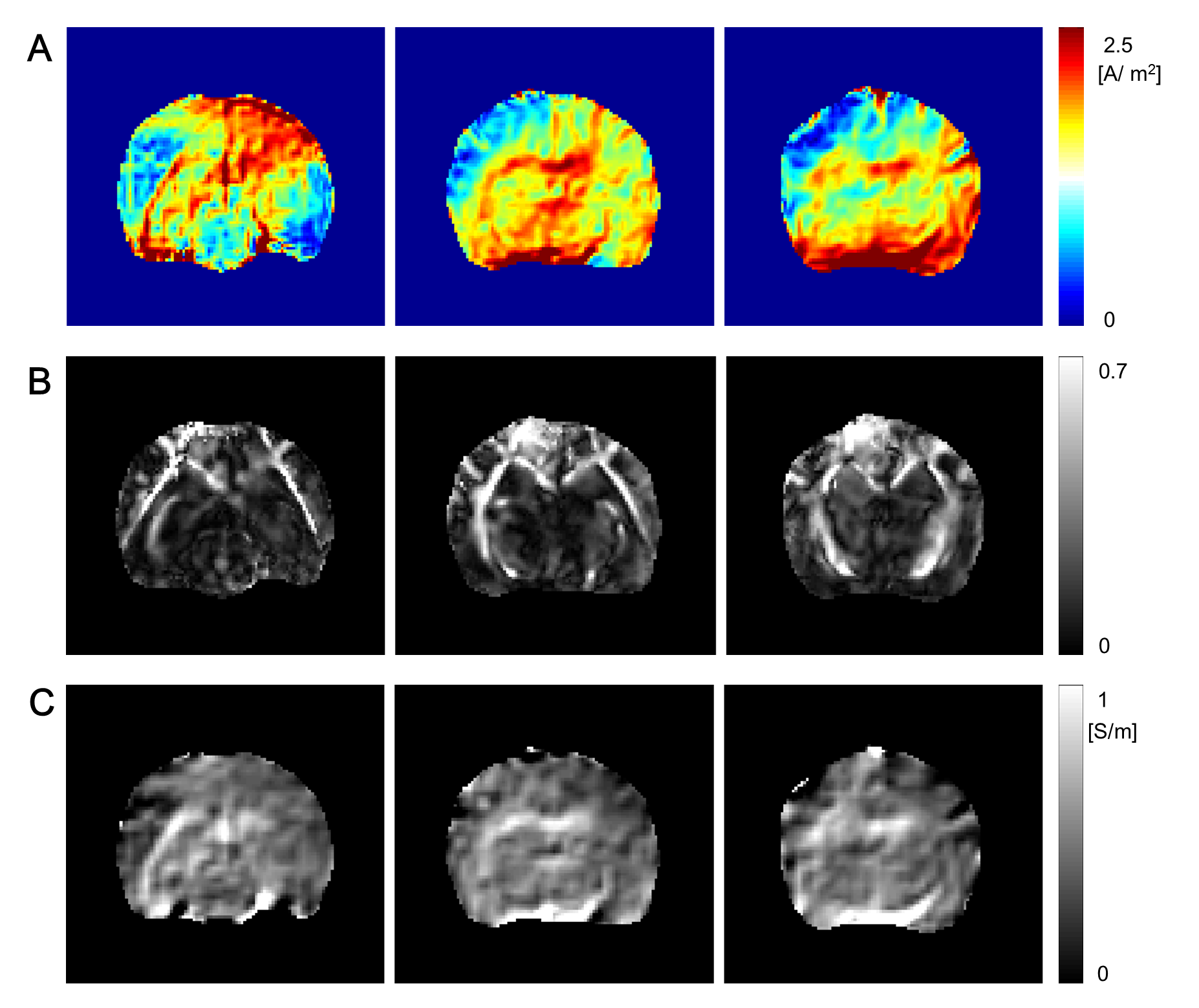

Supplement: S4 Fig — (A) Intensity of the projected current density. (B) Angle images between the second updated velocity vector, D∇u2, and the electric field ∇u. (C) Reconstructed apparent isotropic conductivity images by using J-substitution algorithm. (TIF) [file pone.0197063.s004.tif]
